# Heart–Brain Axis in Viral Myocarditis: Shared Cytokine Pathways, Blood–Brain Barrier Crosstalk, and Neuroinflammatory Consequences

**DOI:** 10.3390/ijms27062603

**Published:** 2026-03-12

**Authors:** Vadim M. Mitrokhin, Andre G. Kamkin, Irina I. Babkina, Irina G. Savinkova, Stanislav A. Shileiko, Roman S. Ovchinnikov, Mitko I. Mladenov

**Affiliations:** 1 Department of Fundamental and Applied Physiology, Russian States Medical University, 117997 Moscow, Russia; mitrokhin_vm@rsmu.ru (V.M.M.); andrey.kamkin@rsmu.ru (A.G.K.); babkina_ii@rsmu.ru (I.I.B.); savinkova_ig@rsmu.ru (I.G.S.); shileiko_sa@rsmu.ru (S.A.S.); ovchinnikov_rs@rsmu.ru (R.S.O.); 2 Institute of Biology, Faculty of Natural Sciences and Mathematics, Ss. Cyril and Methodius University, 1000 Skopje, North Macedonia

**Keywords:** viral myocarditis, heart–brain axis, blood–brain barrier, neuroinflammation, NLRP3 inflammasome, microRNA, cardio-neuroprotection

## Abstract

The heart–brain axis is a bidirectional communication network composed of neural, humoral, and immune pathways that sustain cardiovascular and brain homeostasis. There is mounting evidence that viral myocarditis—a prototype of inflammatory heart disease—acts beyond the myocardium, triggering systemic immune cascades that lead to central nervous system (CNS) involvement. This involvement creates an inflammatory continuum in which cardiac damage and neuroinflammation reinforce each other via common cytokine and molecular mediators. Central mediators in this axis are the proinflammatory cytokines IL-1β, IL-6, tumor necrosis factor (TNF)-α, IL-17, IL-23, and IL-33. These cytokines are released by infected cardiomyocytes and immune cells during myocarditis, inducing endothelial cell (EC) activation, and causing blood–brain barrier (BBB) disruption. Simultaneously, TLR/NF-κB signaling and the stability of endothelial junctions are modulated by regulatory microRNAs such as miR-155 and miR-146a/b, which respectively enhance or attenuate inflammatory signals. Disruption of the BBB allows cytokines and immune cells to enter the brain parenchyma, where they activate microglia and astrocytes through NF-κB-dependent pathways. The resultant neuroinflammation disrupts autonomic equilibrium and leads to sympathetic overdrive, arrhythmogenesis, and overall worsening of cardiac injury, thus forming a self-perpetuating inflammatory cycle between the heart and the brain. Selective modulation of cytokines (anti-IL-1β, IL-6 receptor antagonists, and TNF-α modulators), blockade of the NLRP3 inflammasome, and miRNA therapy (anti-miR-155 and miR-146a mimics) are potential approaches for interrupting the heart–brain inflammatory circuit. In addition, neurotrophic therapies preserving BBB integrity may reduce secondary neuronal damage. Therefore, a future precision cardio-neuroprotective paradigm will rely on the integration of anti-inflammatory, molecular, and neurovascular strategies aimed at limiting systemic cytokine propagation and restoring bidirectional homeostasis through the heart–brain axis.

## 1. Introduction

The heart and the brain are closely connected in a bidirectional manner through a complex network of neural, humoral, and immunological pathways referred to as the heart–brain axis. This mechanism helps maintain cardiac and vascular homeostasis, regulates the autonomic nervous system, and maintains cerebral blood flow. This review first summarizes the mechanisms of cardiac injury in viral myocarditis and then focuses on how these processes extend to the brain through shared inflammatory pathways. Neural elements of this axis consist of vagal and sympathetic fibers that modulate heart rate, contractility, and vascular tone, whereas humoral factors—comprising hormones, cytokines, and extracellular vesicles—facilitate the systemic integration of cardiac and cerebral responses to stress or injury [1,2]. The disruption of this balance may trigger a series of physiological and pathological events; thus, cardiovascular dysfunction is correlated with changes in the central nervous system (CNS) [3].

Inflammatory cytokines generated after heart damage contribute to myocardial remodeling and disrupt the blood–brain barrier (BBB), thereby stimulating glial cells and inducing neuroinflammation [4,5]. These processes are accompanied by activation of inflammasomes, oxidative stress pathways, endothelial adhesion molecules, complement activation, and extracellular vesicle-mediated signaling. Conversely, neuroinflammation and/or cerebrovascular disease can enhance autonomic dysregulation with augmented sympathetic tone and deterioration of cardiac performance [6,7]. This crosstalk forms a self-reinforcing loop in which inflammation in one organ augments the pathological changes in the other, thus emphasizing that the heart–brain axis functions systemically.

In this context, infectious viral myocarditis provides an excellent model for investigating molecular and cellular pathways that link both cardiac and neural inflammation. Viral myocarditis is characterized by viral infection of cardiomyocytes in the acute phase, which activates innate immune sensors such as Toll-like receptors (TLR3, TLR4) and subsequently triggers nuclear factor κB (NF-κB) and interferon-regulatory factors (IRFs) to induce proinflammatory cytokine production, including interleukin (IL)-1β, IL-6, tumor necrosis factor-α (TNF-α), and IL-17 [8,9,10]. These cytokines, which are beneficial for viral eradication, can also induce overreactive immune responses, contributing to myocardial damage and fibrosis and ultimately resulting in long-term cardiac dysfunction [11]. Of note, the systemic production of inflammatory factors can lead to BBB disruption, microglial activation, and secondary neuroinflammation [12,13].

Viral myocarditis is a multi-step disease process that includes direct viral damage, activation of both the innate and adaptive immune systems, and long-term structural changes to the heart [14,15]. Cardiac injury initiates with viral penetration into cardiomyocytes or endothelial cells via specific receptors, including the coxsackievirus–adenovirus receptor (CAR) or angiotensin-converting enzyme 2 (ACE2), followed by intracellular viral replication and cytopathic effects [15,16]. This initial phase leads to cardiomyocyte necrosis and the release of damage-associated molecular patterns (DAMPs) [16].

These DAMPs activate pattern-recognition receptors, such as Toll-like receptors (TLR3, TLR4) and cytosolic RNA/DNA sensors, which initiate innate immune signaling through pathways dependent on MyD88 and TRIF [17]. The activation of NF-κB and interferon-regulatory factors leads to the production of type I interferon and antiviral responses, while also causing inflammatory damage [17,18].

In addition to cytokine production, various molecular injury pathways are initiated:

- Oxidative stress and mitochondrial dysfunction caused by excessive ROS production and compromised mitochondrial metabolism [19].
- Endothelial and microvascular damage, resulting in capillary leakage and compromised myocardial perfusion [20].
- Cell death pathways, such as apoptosis, necroptosis, and pyroptosis, are controlled by caspases, RIPK signaling, and NLRP3 inflammasome activation [17,21].
- Matrix remodeling and fibrosis, instigated by TGF-β signaling and matrix metalloproteinases [22].
- Autoimmune responses, characterized by autoreactive T cells and autoantibodies that target cardiac antigens following viral clearance [14,15,23].

These mechanisms collectively convert an initially protective antiviral response into maladaptive inflammation, myocardial dysfunction, and dilated cardiomyopathy [14,15].

The viral nature of myocarditis is significant given viral infections’ unique combination of direct cytotoxic injury and sustained immune activation. Viral persistence, molecular mimicry, and chronic immune stimulation differentiate viral myocarditis from sterile inflammatory cardiomyopathies and explain its elevated risk of progression to chronic heart failure and systemic inflammatory complications [14,15,23,24].

From a clinical standpoint, patients with myocarditis commonly present with neurological complications such as ischemic or hemorrhagic stroke, encephalopathy, and cognitive impairment. The underlying pathophysiology leading to these presentations is believed to result from hemodynamic instability, cardioembolic phenomena, and cytokine-mediated BBB injury [25]. Indeed, such vascular inflammation appears to promote increased susceptibility to cerebrovascular events and neurodegenerative processes [26,27]; moreover, following recovery from viral myocarditis, patients frequently demonstrate impaired neurocognitive function [28] along with altered autonomic control [29]. Furthermore, the parallel elevation of circulating cytokines, including IL-1β, IL-6, and TNF-α, in both myocardial and cerebral inflammation provides evidence for a common immunopathological network interconnecting these organs [1,30].

Together, these observations emphasize that viral myocarditis is not only a cardiac inflammatory disorder but also a model of systemic inflammation suitable for investigating the molecular and cellular basis of heart–brain crosstalk. Understanding these pathways is important for designing new diagnostic and therapeutic approaches to protect both the heart and the nervous system from inflammation-induced injury.

While substantial mechanistic insight into heart–brain axis signaling in viral myocarditis derives from experimental models (primarily murine CVB3 systems and in vitro neurovascular assays), clinical data in human myocarditis remain comparatively limited. Throughout this review, we clearly distinguish between findings supported by human observational studies or clinical trials and those derived predominantly from preclinical investigations. This distinction is particularly relevant when discussing therapeutic implications.

## 2. Organization of the Heart–Brain Axis

**Figure 1:**
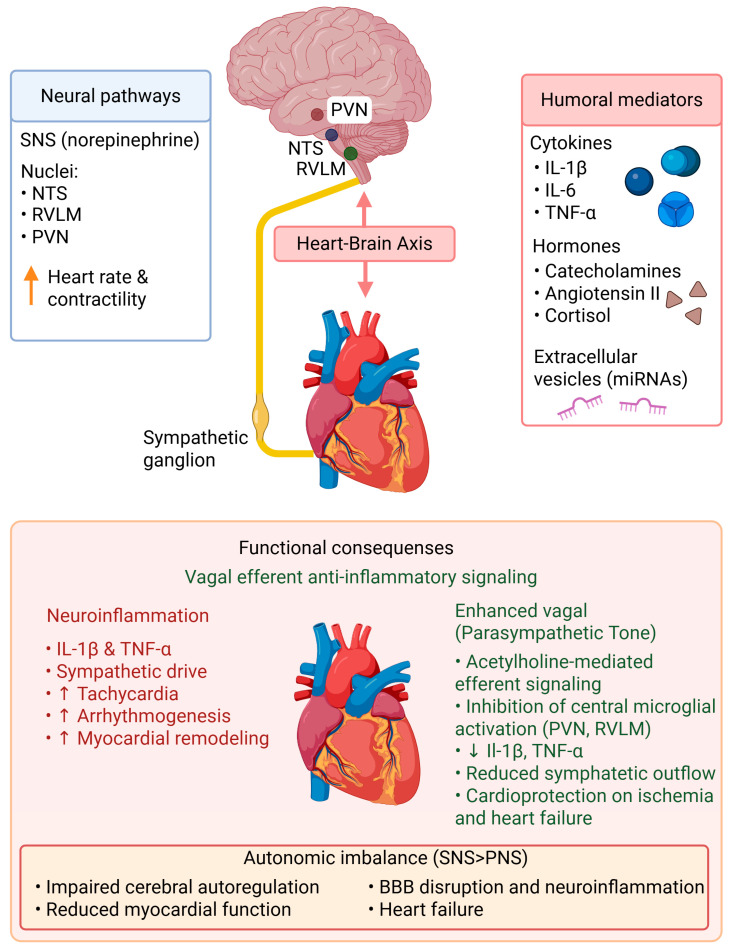
Organization of the heart–brain axis. Schematic illustration of bidirectional communication between the heart and the central nervous system through neural (sympathetic and parasympathetic), humoral (cytokines and hormones), and immune pathways. Central autonomic nuclei regulate cardiac function, while systemic inflammatory mediators and endothelial signaling influence blood–brain barrier integrity and neuroinflammation. (↑-means increase; ↓-means decrease).

The heart–brain axis is an intricate, two-way communication system that links the neural, humoral, and immune systems and affects both the cardiovascular and central nervous systems (CNS). This arrangement ensures that changes in blood flow, pressure, humoral signaling, and metabolic needs are constantly sensed and regulated by the brain. Conversely, neurohumoral responses generated in and released from the CNS have a marked influence on myocardial function, contractility, and vascular tone (Figure 1), [30,31].

### 2.1. Major Mechanistic Pathways of the Heart–Brain Axis

**Figure 2:**
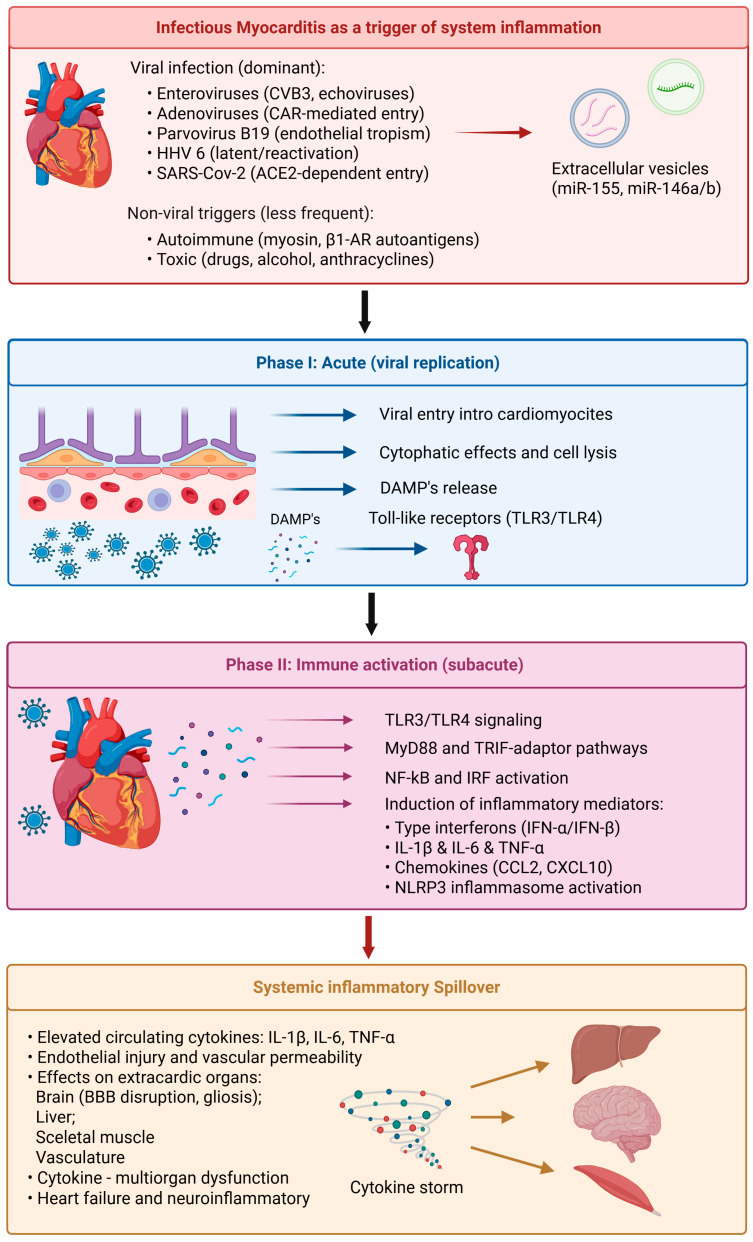
Infectious myocarditis as a trigger of systemic inflammation and multiorgan involvement. Viral infection (e.g., enteroviruses, parvovirus B19, HHV-6, SARS-CoV-2) initiates cardiomyocyte and/or endothelial injury, activating innate immune pathways (e.g., TLR signaling, NF-κB, and the NLRP3 inflammasome). Subsequent cytokine and extracellular vesicle release promotes systemic inflammatory spillover, endothelial dysfunction, and extracardiac organ involvement, including cerebral involvement, thereby linking myocardial inflammation to neuroinflammation and heart failure progression.

The heart–brain axis encompasses multiple interconnected signaling systems, including neural, neuroendocrine, immune, vascular, metabolic, and extracellular vesicle-mediated pathways (Figure 1), [2,32].

(i) Autonomic neural pathway

The most rapid form of heart–brain communication is mediated by sympathetic and parasympathetic circuits that link the central autonomic network (PVN, RVLM, NTS) to the cardiac ganglia [33]. Sympathetic activation increases heart rate, contractility, and arrhythmogenesis, and amplifies inflammatory signaling. Vagal activity, conversely, exerts anti-inflammatory and cardioprotective effects through the cholinergic anti-inflammatory pathway [34,35].

(ii) Neuroendocrine (HPA-axis) signaling

Cardiac injury activates the hypothalamic–pituitary–adrenal (HPA) axis, triggering the release of cortisol, catecholamines, and angiotensin II [36,37,38]. These hormones modulate immune function, alter cerebral perfusion, and enhance sympathetic nervous system activity. Chronic activation leads to adverse cardiac remodeling, blood–brain barrier impairment, and neuroinflammation [37,38,39].

(iii) Immune-inflammatory signaling

Systemic inflammation represents a principal mechanistic link between cardiac injury and CNS dysfunction. Chemokines, cytokines, inflammasome products, and circulating immune cells transmit inflammatory signals to the CNS, where they alter autonomic regulation and activate microglia and astrocytes [2,40].

(iv) Endothelial and blood–brain barrier signaling

The cerebrovascular endothelium serves as a dynamic interface that converts systemic inflammation into neuroinflammatory responses. Endothelial activation, upregulation of adhesion molecules, disruption of tight junctions, and increased permeability facilitate the entry of circulating mediators and immune cells into the CNS [41].

(v) Metabolic and oxidative stress signaling

Cardiac dysfunction causes systemic hypoperfusion, mitochondrial dysfunction, and excessive production of reactive oxygen species. These metabolic disturbances impair central autoregulation, promote neuronal injury, and amplify inflammatory signaling in both organs [10,14].

(vi) Extracellular vesicle and microRNA communication

Extracellular vesicles released from cardiomyocytes, endothelial cells, and immune cells transport proteins, lipids, and regulatory microRNAs to the brain. These vesicles modulate BBB integrity, neuroinflammation, and central autonomic control [42,43,44,45].

These pathways operate concurrently and in a mutually reinforcing manner, forming a cardio-neuro-immune network that is markedly activated during viral myocarditis [2,32].

As illustrated in Figure 1, the nucleus tractus solitarius (NTS), rostral ventrolateral medulla (RVLM), and paraventricular nucleus (PVN) collectively coordinate sympathetic and parasympathetic efferent output, thereby influencing chronotropy, inotropy, and vascular tone. Stress hormones (including catecholamines, angiotensin II, and cortisol), proinflammatory cytokines (including IL-1β, IL-6, and TNF-α), and extracellular vesicles carrying regulatory microRNAs collectively mediate inter-organ signaling. Sustained autonomic imbalance underlies endothelial dysfunction, neuroinflammation, and progressive cardiac remodeling.

### 2.2. Autonomic and Sensory Afferents

Neural connections of the heart–brain axis, encompassing both autonomic efferents and sensory afferent pathways, are primarily autonomic and sensory, respectively. The autonomic nervous system (ANS), consisting of sympathetic and parasympathetic branches, modulates chronotropy, inotropy, and vascular resistance to ensure hemodynamic stability. Sympathetic efferents originating from the rostral ventrolateral medulla traverse the spinal cord to terminate in the cardiac sympathetic ganglia, where norepinephrine release elevates heart rate and contractility [46]. Parasympathetic efferents from the nucleus ambiguus (NA) and dorsal motor nucleus of the vagus nerve (DMVN), conversely, protect the heart and attenuate inflammation by promoting acetylcholine release within cardiac ganglia, reducing cardiac oxygen demand and diminishing systemic inflammation (Figure 1), [34].

Sensory afferent nerves bearing baroreceptors, chemoreceptors, and mechanoreceptors project impulses to the nucleus tractus solitarius (NTS) in the medulla, resulting in reflex regulation of blood pressure and heart rate [33]. This neural feedback loop constitutes an essential cardiorespiratory regulatory circuit for maintaining cardiovascular homeostasis and enabling rapid adaptation to stress, hypoxia, cardiopulmonary challenges, or inflammation. These neural circuits represent the fastest component of the heart–brain axis and provide real-time regulation of cardiovascular and inflammatory responses.

### 2.3. Humoral Mediators: Cytokines, Hormones, and Extracellular Vesicles

Humoral signaling serves as a crucial medium for long-distance communication between the heart and the brain. Circulating cytokines such as IL-1β, IL-6, and TNF-α may cross or modulate the BBB at hypothalamic and brainstem sites of cardiovascular regulation [39,46]. These cytokines, when present in the brain, also activate endothelial and glial cells, leading to altered neurovascular function and driving neuroinflammatory cascades that exert feedback onto the cardiovascular system.

In viral myocarditis, humoral immunity includes not only soluble cytokines and hormones but also adaptive immune mediators such as immunoglobulins and complement components. Under physiological conditions, large circulating antibodies (e.g., IgG, IgM) cannot traverse the intact blood–brain barrier due to restricted paracellular permeability at tight junctions and limited transcytotic transport [47,48]. During systemic inflammation or cytokine-induced endothelial activation, BBB permeability increases via NF-κB- and RhoA/ROCK-dependent mechanisms, facilitating immunoglobulin extravasation into the CNS parenchyma [49,50]. Furthermore, once the barrier is compromised, Fc receptor-mediated transport and complement activation may further amplify neuroinflammatory responses. Autoantibodies directed against cardiac antigens (e.g., β1-adrenergic receptors or myosin) have been described in viral myocarditis and may contribute indirectly to neurovascular dysfunction through systemic immune activation.

Exosomes (30–150 nm), microvesicles (100–1000 nm), and apoptotic bodies constitute the major types of extracellular vesicles (EVs), each distinguished by distinct biogenesis pathways and cargo-selection mechanisms (Figure 1). Exosomes arise from multivesicular bodies through ESCRT-dependent or -independent pathways, whereas microvesicles directly bud from the plasma membrane [51]. During viral myocarditis, stressed cardiomyocytes, endothelial cells, and infiltrating immune cells release extracellular vesicles enriched in proinflammatory cytokines, mitochondrial DNA, and regulatory microRNAs such as miR-155 and miR-146a/b. EVs interact with the blood–brain barrier through receptor-mediated endocytosis, membrane fusion, or caveolin-dependent uptake. Experimental evidence demonstrates that systemically derived inflammatory EVs can cross or alter BBB function, thereby destabilizing endothelial tight junctions and activating microglia [51,52]. Accordingly, EVs are not merely passive carriers; rather, they function as structured intercellular signaling platforms that propagate cardiac inflammatory signals to the CNS, establishing a mechanistic link between myocardial injury and central neuroinflammation.

#### 2.3.1. Mechanisms of Cytokine Transmission Across the Heart–Brain Axis

Circulating cytokines influence the brain through several complementary mechanisms:

1. Direct endothelial cell signaling: IL-1β, IL-6, and TNF-α bind to their receptors on cerebrovascular endothelial cells, activating the NF-κB and JAK/STAT3 pathways. This promotes disassembly of tight junctions, upregulation of ICAM-1 and VCAM-1, and enhanced leukocyte transmigration [47,49].
2. Circumventricular organ access represents another route, as areas devoid of a conventional blood–brain barrier (e.g., area postrema, subfornical organ) facilitate cytokine detection and transmission to hypothalamic and autonomic nuclei, thereby altering neuroendocrine and sympathetic responses [48].
3. Neural afferent activation occurs when peripheral cytokines engage vagal afferent fibers, transmitting inflammatory signals to the nucleus tractus solitarius, which modulates cardiac and autonomic function [48].
4. Saturable transport systems provide an additional regulated pathway, whereby certain cytokines, including IL-1 and TNF-α, are actively transported across the BBB, enabling peripheral immune-to-brain signaling [47].

These interconnected mechanisms illustrate how cardiac inflammation propagates autonomic dysregulation and neuroinflammation within the CNS.

#### 2.3.2. Cellular and Molecular Mechanisms of Neuroinflammation

Neuroinflammation is a coordinated response of the neurovascular unit to systemic or local immune activation, involving complex interactions between microglia, astrocytes, endothelial cells, infiltrating leukocytes, and neurons. Although initially protective, this response, if persistent, leads to neuronal dysfunction and autonomic dysregulation.

Microglial activation. Microglia are the CNS’s resident innate immune cells. Circulating cytokines (IL-1β, IL-6, TNF-α) or pathogen-associated molecular patterns (PAMPs) engage microglial Toll-like receptors and inflammasome complexes, particularly NLRP3, promoting IL-1β maturation and augmenting NF-κB signaling [53,54]. Upon activation, microglia may polarize toward a proinflammatory phenotype, producing reactive oxygen species (ROS), nitric oxide, and excitotoxic mediators.

Astrocyte reactivity. Astrocytes undergo phenotypic changes in response to cytokines. Proinflammatory signaling induces A1-type astrocytic activation, associated with upregulation of complement components and reduction in neurotrophic support [55]. Astrocytes also regulate glutamate homeostasis and BBB stability; their dysfunction promotes excitotoxicity and barrier instability.

Endothelial and BBB remodeling. Neuroinflammation disrupts endothelial cytoskeletal organization, promotes tight junction degradation, and enhances leukocyte adhesion. NF-κB-mediated upregulation of ICAM-1 and VCAM-1 facilitates immune cell diapedesis, while RhoA/ROCK signaling drives actin stress fiber formation and paracellular leakage [56].

Peripheral immune cell recruitment. BBB disruption enables circulating monocytes and Th17 lymphocytes to enter the CNS, sustaining IL-17- and IL-23-driven inflammatory cascades [57]. This infiltration links systemic myocarditis-associated inflammation to central immune amplification.

Neuronal dysfunction and autonomic imbalance. Sustained exposure to IL-1β and TNF-α alters synaptic transmission, increases glutamatergic neuronal excitability, and disrupts autonomic centers in the hypothalamus and brainstem. Collectively, these changes drive sympathetic hyperactivation and reduced vagal tone, exacerbating cardiac injury [53].

The functional significance of neuroinflammation is context-dependent. During acute infection, it may contribute to antiviral defense and tissue protection. However, chronic low-grade activation promotes neurodegeneration, microvascular rarefaction, and long-term autonomic dysregulation. Therefore, in viral myocarditis, neuroinflammation should be conceptualized as a dynamic, multicellular process rather than a static inflammatory state.

### 2.4. Impact of Neuroinflammation on the Cardiac Autonomic Control

As a central amplification mechanism within the heart–brain axis, neuroinflammation powerfully modifies cardiac autonomic control, largely through perturbation of sympathetic and parasympathetic tone. Localized microglial activation in brain regions, including the paraventricular nucleus (PVN) and rostral ventrolateral medulla (RVLM), results in overproduction of proinflammatory cytokines such as IL-1β and TNF-α, subsequently leading to an increase in neuronal excitability and sympathetic output [36,40]. Sustained activity of these central circuits results in sympathetic hyperactivation, tachycardia, arrhythmogenesis, and adverse myocardial remodeling [37]. Conversely, enhancement of vagal tone attenuates CNS inflammation and counteracts heart failure and ischemia in experimental animal models (Figure 1), [36].

### 2.5. Pathophysiological Crosstalk Between Myocardial Ischemia and Cerebrovascular Injury

Ischemic injury exemplifies the clinical manifestation of heart–brain axis dysregulation. The heart and brain maintain a fundamentally reciprocal relationship, which is most dramatically demonstrated in the context of ischemic injury. Systemically, myocardial ischemia leads to inflammatory responses and increased catecholamine levels, which impair cerebrovascular autoregulation and contribute to BBB disruption [32].

Cerebrovascular complications related to myocarditis and systemic inflammation arise through multiple mechanisms, including vascular inflammation, endothelial dysfunction, coagulopathy, and hemodynamic instability. Elevated circulating cytokines, including IL-6 and TNF-α, upregulate endothelial activation, increase tissue factor expression, and impair fibrinolysis, collectively promoting a prothrombotic state [58,59]. Systemic inflammation further enhances platelet reactivity and promotes neutrophil extracellular trap (NET) formation, thereby amplifying thromboembolic risk.

Endothelial injury impairs cerebrovascular autoregulation. Cytokines, oxidative stress, and reduced endothelial nitric oxide synthase (eNOS) activity compromise nitric oxide bioavailability, attenuating vasodilatory responses and increasing the risk of ischemic injury [60]. Microvascular dysfunction may precede overt cerebrovascular events, manifesting as cerebral hypoperfusion or small-vessel disease.

In myocarditis, cardioembolic mechanisms represent a critical pathway of cerebral injury, whereby intracardiac thrombus formation enables embolization to the cerebral vasculature. Patients with ventricular systolic dysfunction, regional wall motion abnormalities, intracardiac thrombus formation, or arrhythmias, such as atrial fibrillation, carry an elevated risk of embolic stroke [31]. Systemic hypotension and cardiogenic shock may precipitate watershed infarcts and hypoxic–ischemic encephalopathy through reduction in cerebral perfusion pressure.

BBB disruption and altered cerebrovascular hemodynamics during systemic inflammation create conditions predisposing to hemorrhagic complications. Elevated matrix metalloproteinase activity and endothelium-destabilizing cytokines increase the likelihood of hemorrhagic transformation following ischemic events [61].

Cerebrovascular injury further amplifies systemic inflammation through the release of damage-associated molecular patterns (DAMPs), which activate peripheral immune cells and exacerbate cardiac injury. Therefore, cerebrovascular events can both precipitate and perpetuate heart–brain axis dysfunction.

Conversely, cerebrovascular events, including stroke, can lead to myocardial dysfunction—referred to as stroke–heart syndrome—manifesting as arrhythmias, reduced ejection fraction, and elevated troponin levels [41]. This bidirectional susceptibility is explained by a common profile of inflammatory mediators (IL-6, TNF-α) and neurohumoral and autonomic activation, which ultimately link cerebral and cardiac ischemic injury through a self-perpetuating cycle (Figure 1), [25,31].

Collectively, these neural and humoral mechanisms demonstrate the integrative nature of the heart–brain axis. Its dysregulation during myocarditis, ischemia, or systemic inflammation underlies injury to secondary organs, underscoring the importance of integrated treatment strategies targeting both cardiac and neural pathways.

## 3. Infectious Myocarditis as a Trigger of Systemic Inflammation

Infectious myocarditis is an inflammatory myocardial disease that occurs primarily due to viral infection, although autoimmunity and toxicity-associated mechanisms can also be involved. The disease serves as a paradigmatic model of localized cardiac injury that transforms into a systemic inflammatory syndrome, connecting the heart and extracardiac organs—such as the brain—via common cytokine-mediated signaling pathways (Figure 2), [24].

### 3.1. Etiology and Classification

Etiologically, myocarditis can be divided into infectious, autoimmune, and toxic types.

Clinical subtypes:

○ Infectious myocarditis, predominantly viral in origin, represents the majority of cases in both clinical and experimental settings.
○ Autoimmune myocarditis develops when self-reactive T lymphocytes and autoantibodies recognize myocardial antigens (e.g., cardiac myosin, β1-adrenergic receptors), typically following viral or bacterial exposure.
○ Toxic myocarditis can result from exposure to drugs, alcohol, or chemotherapeutic agents (e.g., anthracyclines), which induce direct cardiomyocyte injury and sterile inflammation [15].

Viruses are the most common infectious agents, but myocarditis can also be induced by bacteria (e.g., *Borrelia burgdorferi*), parasites (e.g., *Trypanosoma cruzi*), and fungi, particularly in specific geographic regions and among immunocompromised patients.

### 3.2. Specific Viruses in Myocarditis

Viral myocarditis is predominantly caused by cardiotropic viruses capable of infecting cardiac cells and replicating (Table 1).

The major viral groups include:

○ Enteroviruses (e.g., Coxsackievirus B3 and echoviruses): historically recognized pathogens that induce rapid cardiomyocyte cytolysis as a result of viral protease activity, followed by immune-mediated injury (Table 1), [16].
○ Adenoviruses (types 2 and 5), which enter cardiomyocytes through a coxsackievirus–adenovirus receptor (CAR)-mediated mechanism, leading to activation of innate immune signaling (Table 1), [62].
○ Parvovirus B19, which does not directly infect cardiac myocytes but instead targets endothelial cells, inducing microvascular inflammation and subsequent endothelial dysfunction with secondary myocyte injury (Table 1), [20].
○ Human herpesvirus 6 (HHV-6), which infects cardiac tissue latently and may be reactivated under stress or immunosuppression, leading to persistent inflammation (Table 1), [63].
○ Severe Acute Respiratory Syndrome Coronavirus 2 (SARS-CoV-2), which is a recognized cause of myocarditis due to Angiotensin-Converting Enzyme 2 (ACE2)-dependent viral entry, endothelial injury, and dysregulation of the immune response, resulting in multisystem inflammatory syndrome (Table 1), [64,65].

Although these viruses share the same downstream inflammatory pathways, including NF-κB activation, inflammasome signaling, and cytokine amplification, they differ considerably in their cellular targets and mechanisms of immune activation. Enteroviruses, including CVB3, generally induce direct cytolysis of cardiomyocytes, subsequently leading to adaptive immune amplification [16]. Conversely, parvovirus B19 primarily affects endothelial cells, resulting in microvascular inflammation and endothelial dysfunction rather than widespread myocyte necrosis. SARS-CoV-2-associated myocarditis is marked by endothelial damage, microthrombosis, and dysregulated innate immunity, frequently occurring within a multisystem inflammatory framework (Table 1), [65]. Consequently, although convergent cytokine pathways link these etiologies to heart–brain axis activation, the upstream triggers and vascular versus myocyte-dominant injury patterns exhibit considerable variability.

### 3.3. Phases of the Disease

Viral myocarditis follows a typical pattern of three sequential but overlapping phases, each characterized by specific molecular and cellular processes [19]:

(i) Acute phase (viral replication): viral entry into cardiomyocytes, followed by cytopathic effects and cell lysis, occurs concurrently with Damage-Associated Molecular Patterns (DAMP) release. These signals trigger Toll-like receptors (TLRs) and innate immune cascades within infected and neighboring cells (Figure 2).
(ii) Subacute or immune activation phase: activation of TLR3 or TLR4 leads to downstream adaptor signaling through Myeloid Differentiation Primary Response 88 (MyD88) and TIR-domain-containing adaptor inducing interferon-β (TRIF), resulting in activation of NF-κB and IRFs [8]. These transcription factors induce the production of type I interferons (IFN-α/β) and proinflammatory cytokines (IL-1β, IL-6, TNF-α), which are protective against viral infection but also exacerbate tissue inflammation [8]. During this phase, cytotoxic CD8^+^ T cells and macrophages infiltrate the heart and directly target infected myocytes, leading to nonspecific bystander tissue loss (Figure 2).
(iii) Chronic phase (remodeling and dilated cardiomyopathy): when viral clearance is incomplete or autoimmune activation persists, chronic inflammation with fibroblast activation and extracellular matrix (ECM) remodeling occurs, which eventually results in dilated cardiomyopathy (DCM) characterized by ventricular dilatation, wall thinning, and progressive contractile impairment (Figure 2), [66].

### 3.4. Immune Signaling Cascades and Cytokine Amplification

Immune activation in viral myocarditis initially represents a protective antiviral response that may progress into a maladaptive cytokine storm if dysregulated.

○ TLR3/4 signaling triggers MyD88- and TRIF-mediated activation of NF-κB and IRF pathways to generate inflammatory mediators [IL-1β, IL-6, TNF-α, and chemokines (e.g., C–C motif chemokine ligand 2 (CCL2) and C–X–C motif chemokine ligand 10 (CXCL10)], which further attract immune cells to the myocardium [17].
○ The NLRP3 inflammasome is engaged, leading to enhanced IL-1β activation and pyroptotic cell death [21].
○ Sustained cytokine levels and oxidative stress damage the endothelial lining, leading to increased vascular permeability and systemic inflammation [22].

These inflammatory responses not only exacerbate myocardial damage but also spread to other organs, including the brain, liver, and skeletal muscle, via circulating cytokines and extracellular vesicles.

### 3.5. Progression from Local to Systemic Inflammation

Initially confined to the myocardium, inflammation in viral myocarditis typically evolves into a systemic cytokine storm, particularly during severe or fulminant disease. Elevated levels of circulating IL-6, TNF-α, and IL-1β are detected rapidly and are associated with cardiac dysfunction and multiorgan failure [14]. These cytokines increase BBB permeability and induce microglial activation, thus connecting cardiac inflammation to secondary neuroinflammatory events [5].

Systemic spillover of these mediators also precipitates metabolic and vascular dysfunction, contributing to coagulopathy, heart failure, and neuromuscular injury. In this context, viral myocarditis serves as a paradigm in which localized viral invasion leads to subsequent systemic and cerebral immunologic disruption.

Clinical observations suggest that the disease course deviates considerably from the assumption of a linear progression from viral entry to immune-mediated injury. For instance, parvovirus B19-associated myocarditis frequently demonstrates endothelial-predominant involvement with minimal myocyte necrosis, while CVB3 models are characterized by direct cytolysis followed by adaptive immune amplification (Table 1). These distinctions have therapeutic implications; antiviral strategies may be pertinent in early cytolytic phases but less efficacious in immune-mediated or virus-negative inflammatory cardiomyopathy. Moreover, the majority of mechanistic insights originate from murine models, which inadequately replicate the immunophenotypic diversity observed in human cohorts. Therefore, caution is warranted when extrapolating findings from experimental myocarditis models to clinical heart–brain axis dysfunction.

## 4. Cytokine Mediators in Heart–Brain Crosstalk

While the cytokine networks described below are activated in viral myocarditis, many of these inflammatory mediators also operate in non-viral inflammatory cardiomyopathies; therefore, mechanistic overlap should not be interpreted as viral specificity without etiologic confirmation.

Both cytokines and microRNAs (miRNAs) play important roles in mediating heart–brain communication during inflammation, including in viral myocarditis. These molecules not only regulate immune activation and cardiac remodeling but also affect BBB integrity and neuroinflammation. This crosstalk occurs through circulating cytokines, extracellular vesicles, and signaling intermediates that link myocardial inflammation to central nervous system (CNS) dysfunction [14,67,68].

### 4.1. IL-1β/NLRP3 Inflammasome Axis

The NLRP3 inflammasome acts as a central signaling platform connecting myocardial inflammation and neuroinflammation. Its activation occurs in macrophages, fibroblasts, and cardiomyocytes, where it enhances caspase-1-dependent cleavage of pro-IL-1β and pro-IL-18 into their active forms [21]. Activation occurs in two stages: (1) NF-κB-dependent priming, initiated by Toll-like receptor (TLR) engagement or cytokine stimulation; and (2) assembly and activation via mitochondrial reactive oxygen species (ROS), potassium efflux, or calcium flux (Table 2), [69].

Coxsackievirus B3 (CVB3) capsid proteins serve as pathogen-associated molecular patterns (PAMPs) and can trigger NLRP3 oligomerization and IL-1β release, contributing to the development of myocarditis, which is often characterized by excessive inflammation, fibroblast proliferation, and interstitial fibrosis [70,71]. Persistent inflammasome activation further promotes myocardial necrosis, resulting in dilated cardiomyopathy (DCM).

In the brain, IL-1β and TNF-α impair tight junction (TJ) protein expression (claudin-5, occludin, and Zonula occludens-1 (ZO-1)) at the BBB, leading to increased permeability and immune cell influx [4]. These cytokines also activate astrocytes and microglia through an NF-κB-dependent mechanism, leading to gliosis, oxidative stress, and neuronal injury [72]. Sustained elevation of IL-1β is associated with chronic neuroinflammation linked to cognitive decline and neurodegenerative diseases following systemic inflammatory conditions (Table 2), [73].

### 4.2. IL-6 Signaling

IL-6 mediates a variety of context-specific effects via two main signaling pathways (Table 2):

○ Classical signaling occurs through interaction with the membrane-bound interleukin-6 receptors (IL-6R) and coreceptor Glycoprotein 130 (GP130), predominantly in hepatocytes and immune cells.
○ Trans-signaling is mediated by soluble interleukin-6 receptor (sIL-6R)-bound IL-6, enabling the cytokine to exert effects on cells lacking IL-6R, such as endothelial and glial cells [74,75].

In viral myocarditis, IL-6 is important for acute antiviral response through the induction of acute-phase proteins and T cell differentiation. However, in later stages, persistent upregulation of IL-6 drives fibroblast activation, leading to ventricular remodeling and heart failure (Table 2), [76,77].

At the BBB, IL-6 increases endothelial permeability through Janus Kinase/Signal Transducer and Activator of Transcription 3 (JAK/STAT3)-mediated reduction in occludin and claudin-5, resulting in tight junction disruption [78]. In the CNS, IL-6 influences astrocyte–microglia interplay by increasing glial activation and neuroinflammatory sensitization. It has been associated with neuronal apoptosis and cognitive impairment in animal models via IL-6 overactivation, highlighting its bidirectional cardiocerebral effects (Table 2), [79].

### 4.3. TNF-α

Tumor necrosis factor-α (TNF-α) is a central proinflammatory cytokine with dual harmful and protective effects depending on receptor binding. Apoptotic and necroptotic cascades occur via caspase-8 during Tumor Necrosis Factor Receptor 1 (TNFR1) stimulation, whereas survival and tissue repair in affected cells are promoted by TNFR2 signaling, which activates NF-κB and Phosphoinositide 3-Kinase/Protein Kinase B (PI3K/Akt) signaling pathway (Table 2), [80].

In the myocardium, TNF-α increases the expression of endothelial adhesion molecules (Intercellular Adhesion Molecule 1 (ICAM-1), and Vascular Cell Adhesion Molecule 1 (VCAM-1)), promotes leukocyte accumulation, and induces matrix metalloproteinases (MMP-2, MMP-9), leading to extracellular matrix degradation and adverse remodeling (Table 2), [81].

In the CNS, TNF-α mediates BBB disruption through Ras homolog family member A/Rho-associated protein kinase signaling pathway (RhoA/ROCK)-dependent actin cytoskeletal contraction and inhibition of Wnt/β-catenin signaling, resulting in endothelial destabilization [82]. It also triggers astrocytes to transform into the A1 neurotoxic phenotype and increases glutamate release, neuronal excitotoxicity, and oxidative injury [83]. These findings collectively identify TNF-α as a crucial molecule connecting cardiac inflammation to neuroinflammatory damage (Table 2).

### 4.4. IL-17/IL-23 Axis

There is a key role for the IL-17/IL-23 axis in T helper 17 (Th17) cell-mediated autoimmunity and chronic inflammation. In viral myocarditis, Th17 cells infiltrate the myocardium, and IL-17A promotes cardiac neutrophil accumulation, myocyte necrosis, and fibrotic matrix remodeling [84]. IL-23, produced by macrophages and dendritic cells, enhances Th17 differentiation and maintains IL-17 secretion via the STAT3 cascade (Table 2), [85,86].

Experimental models of CVB3 myocarditis show that IL-17A knockout or inhibition of IL-23 dramatically reduces cardiac infiltrates and damage [87]. Outside the heart, IL-17 and IL-23 mediate BBB breakdown via mechanisms involving the promotion of endothelial tight-junction disassembly and induction of glial cell activation. Increased IL-17 also promotes microglial-derived IL-1β, leading to secondary brain injury and cognitive deficit [88]. In this manner, these two cytokines serve as systemic inflammatory amplifiers that connect myocarditis to neuroinflammation (Table 2).

### 4.5. IL-33

Interleukin-33 (IL-33) plays a dual role in myocardial and cerebral inflammation, depending on the disease setting and signal strength. By interacting with the receptor ST2L, IL-33 initiates the MyD88/TRAF6/NF-κB pathway and triggers both protective and proinflammatory signaling (Table 2), [89].

In the heart, IL-33 can reduce fibrosis and apoptosis by enhancing Th2 cytokines (IL-4, IL-13) and decreasing cardiomyocyte hypertrophy. However, chronically elevated IL-33 may exacerbate inflammation by activating mast cells and eosinophils, ultimately causing tissue damage [90]. In the CNS, IL-33 is released from astrocytes and endothelial cells following injury and stimulates microglial activation as well as cytokine release (IL-6, IL-8). These mediators further enhance BBB permeability, resulting in neurovascular dysfunction (Table 2), [91].

## 5. MicroRNA Regulation of Heart–Brain Inflammatory Signaling

MicroRNAs (miRNAs) are small non-coding RNAs (~22 nucleotides) that regulate gene expression post-transcriptionally through mRNA degradation or translational repression. Unlike cytokines, which transduce signals via extracellular receptor binding, miRNAs operate intracellularly or through intercellular transfer via extracellular vesicles, thereby modulating entire signaling networks rather than individual pathways.

Over 2000 human miRNAs have been identified; however, only a subset is consistently associated with inflammatory cardiomyopathies and neuroinflammatory processes. Viral myocarditis and systemic inflammation induce coordinated alterations in the miRNA expression profiles of the heart and brain, some of which are known to participate in both cardiac remodeling and regulation of blood–brain barrier integrity (Table 3).

MicroRNAs (miRNAs) act as post-transcriptional regulators of inflammation and play key roles in myocardial and neuroinflammatory signaling. Of these, miR-155 and miR-146a/b have been identified as central molecules involved in the regulation of TLR and NF-κB pathways (Table 3), [92].

### 5.1. miR-155 (Pro-Inflammatory Amplifier)

**Figure 3:**
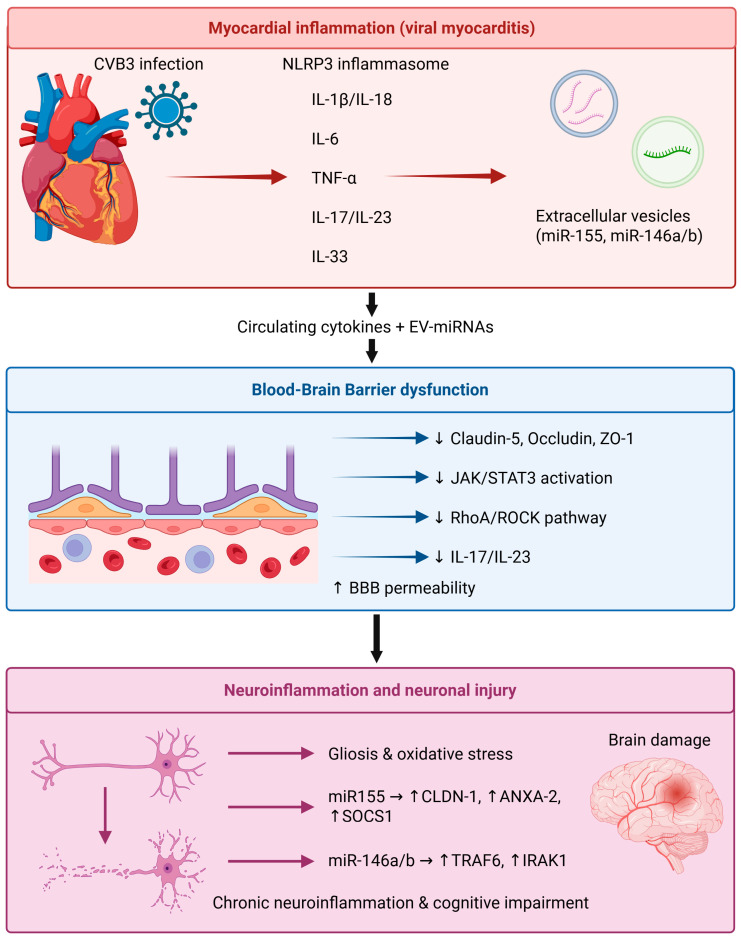
Cytokine- and microRNA-mediated heart–brain crosstalk during viral myocarditis. Myocardial inflammation activates proinflammatory cytokine pathways (IL-1β, IL-6, TNF-α, IL-17/IL-23) and promotes the release of extracellular vesicles containing regulatory microRNAs (e.g., miR-155, miR-146a/b). These mediators impair blood–brain barrier integrity, activate glial cells, and induce neuroinflammation, contributing to autonomic imbalance and adverse cardiac remodeling. (↑-means increase; ↓-means decrease).

miR-155 functions as an immune-stimulatory amplifier that directly targets tight junction (TJ)-associated molecules Claudin-1 (CLDN-1) and Annexin A2 (ANXA-2), while also downregulating suppressor of cytokine signaling 1 (SOCS1), thereby permitting persistent cytokine signaling and sustained activation of macrophages (Table 3), [93]. Furthermore, high-level expression of miR-155 is associated with exacerbated myocarditis and cardiac dysfunction, as well as impaired endothelial function and BBB disruption (Figure 3).

### 5.2. miR-146a/b (Negative Feedback Regulator)

miR-146a/b act in a context-dependent, dual manner (Table 3). They induce anti-inflammatory functions by targeting the adaptor proteins TRAF6 and Interleukin-1 Receptor-Associated Kinase 1 (IRAK1) in the NF-κB pathway but can also favor Th17 differentiation through Retinoic acid-related Orphan Receptor gamma t (RORγt) regulation under chronic stimulation (Figure 3), [94,95].

### 5.3. miR-21 (Fibrosis and Neurovascular Remodeling)

During myocardial injury, miR-21 is significantly upregulated and facilitates cardiac fibroblast activation by targeting SPRY1 and PTEN, thereby enhancing MAPK and Akt signaling [96]. In the central nervous system, miR-21 regulates astrocyte reactivity and contributes to gliosis. In inflammatory conditions, elevated miR-21 levels are linked to BBB disruption and microvascular remodeling [97]. Because miR-21 participates in both fibrosis and neurovascular plasticity, it serves as a molecular link between chronic myocardial remodeling and chronic neuroinflammation.

### 5.4. miR-126 (Endothelial Stability Regulator)

miR-126 is highly abundant in endothelial cells and preserves vascular integrity by modulating VCAM-1 expression and PI3K signaling [98]. Decreased miR-126 levels during systemic inflammation impair endothelial repair and increase vascular permeability. In the context of myocarditis, the dysregulation of miR-126 may contribute to both coronary microvascular dysfunction and the destabilization of the blood–brain barrier (BBB).

### 5.5. miR-223 (Inflammasome Modulation)

miR-223 directly targets NLRP3 transcripts to suppress the activation of the NLRP3 inflammasome [99]. Reduced miR-223 levels have been linked to elevated IL-1β production in inflammatory heart disease. In microglia, miR-223 inhibits excessive inflammasome activation, indicating that its downregulation may promote prolonged neuroinflammation during systemic immune activation.

While many other microRNAs (miRNAs) are involved in regulating inflammation, the examples listed above represent well-characterized mediators that have been shown to participate in myocardial inflammation, endothelial dysfunction, and neuroinflammatory signaling. Future multi-omics approaches are expected to expand the catalog of miRNAs associated with heart–brain axis dysregulation.

## 6. Blood–Brain Barrier: Central Node of Heart–Brain Crosstalk

The blood–brain barrier (BBB) is a key regulator of central nervous system (CNS) homeostasis and acts as the major site where systemic inflammatory signals modulate neural function. In the setting of myocarditis and systemic inflammation, the BBB serves as a critical interface for reciprocal heart–brain communication, translating localized cardiac injury into neuroinflammation and cognitive dysfunction [100].

Under normal conditions, the BBB effectively restricts immunoglobulin passage, thereby preserving CNS immune privilege [49]. However, cytokine-induced endothelial contraction, RhoA/ROCK activation, and matrix metalloproteinase-mediated basement membrane degradation permit antibody extravasation from the circulation [49,50]. Upon entry into the CNS, IgG complexes activate Fcγ receptors on microglia and complement cascades, thereby exacerbating neuroinflammatory injury and perpetuating glial activation [48].

### 6.1. Structure and Function of the Neurovascular Unit

The BBB is an integral component of the neurovascular unit (NVU) comprising endothelial cells, pericytes, astrocytic end-feet, microglia, and neurons, which communicate with each other to maintain the transport of ions, nutrients, and signaling molecules between the circulation and brain parenchyma [101].

○ Endothelial cells form tight junctions composed of claudin-5, occludin, and ZO-1 to restrict paracellular diffusion.
○ Pericytes, located adjacent to endothelial cells and embedded within the basement membrane, maintain capillary integrity and regulate endothelial cell stability and vascular tone via Platelet-Derived Growth Factor beta (PDGF-β) and Transforming Growth Factor beta (TGF-β) signaling [102,103].
○ Astrocytic end-foot processes cover >90% of the cerebral microvasculature and contribute to BBB stabilization by releasing angiopoietin-1 (Ang-1) and glial cell line-derived neurotrophic factor [102,103].

Disruption of this spatial coordination compromises BBB selectivity and allows the entry of circulating cytokines, inflammatory cells, and microbial products—all of which are frequently elevated during myocarditis and systemic inflammation.

### 6.2. Mechanisms of Cytokine-Induced Permeability

Inflammatory cytokines derived from cardiac injury have profound effects on BBB permeability and transport mechanisms, leading to the progressive development of endothelial dysfunction and neuroinflammation.

○ IL-1β and TNF-α promote endothelial activation and leukocyte adhesion, facilitating immune cell transmigration into the CNS [104,105]. These effects are mediated through inflammatory signaling pathways that destabilize cytoskeletal architecture and compromise tight junction integrity.
○ IL-6 further contributes to barrier instability by altering tight junction organization and increasing paracellular permeability [106].
○ IL-17 and IL-23 enhance endothelial permeability through both transcellular and cytoskeletal mechanisms, amplifying barrier dysfunction [88].
○ microRNA-155 (miR-155) adversely affects BBB integrity by downregulating tight junction protein CLDN-1 and adhesion molecule ANXA-2, both of which are essential structural components for maintaining endothelial barrier integrity [107]. Elevated miR-155 levels in circulating exosomes during viral myocarditis may further contribute to increased endothelial permeability and prolonged neuroinflammation [108].

Together, these processes represent a multifaceted inflammatory attack on the BBB, converting it from a selective permeability barrier into a conduit for peripheral immunoinflammatory mediators.

### 6.3. Chronic Neuroinflammation and Heart–Brain Interactions

When the BBB is disrupted, it functions not only as a passive filter but also as an active amplifier of systemic inflammation. The weakened barrier enables circulating cytokines, monocytes, and activated lymphocytes to gain access to the CNS, where they engage with resident microglia and astrocytes, thereby sustaining neuroinflammation [79].

Activated glial cells amplify inflammatory signaling and further compromise barrier stability, creating a self-perpetuating cardio–neuroinflammatory cycle.

This chronic inflammatory dialogue may present clinically as encephalopathy, autonomic dysregulation, cognitive decline, or, in severe cases, neurodegeneration. The BBB represents a key anatomical structure mediating cardiac-driven immune activation and progressive CNS injury, highlighting it as a potential therapeutic target for modulating heart–brain inflammatory crosstalk [41].

It is crucial to understand that BBB dysfunction in systemic inflammation does not equate to uniform structural degradation. Emerging evidence points to a range of changes, from subtle alterations in transporter expression and transcellular trafficking to overt disassembly of tight junctions. Furthermore, the regional vulnerability of the hippocampal, hypothalamic, and brainstem nuclei varies, which could explain the differential cognitive and autonomic outcomes. These subtleties emphasize that BBB disruption in myocarditis is likely context-dependent rather than a dichotomous pathological phenomenon.

## 7. Therapeutic Implications and Future Perspectives

Therapeutic approaches may vary significantly between viral-positive and virus-negative (autoimmune) myocarditis. In active viral myocarditis, preserving antiviral immunity is essential, and indiscriminate immunosuppression could impair viral clearance. Conversely, immunosuppressive therapy has been shown to benefit patients with biopsy-proven virus-negative inflammatory cardiomyopathy. Consequently, before initiating cytokine-targeted therapies such as IL-1 or IL-6 inhibition, etiologic stratification and viral genome assessment are warranted. Therefore, precise phenotyping is essential for the rational modulation of the heart–brain axis in myocarditis.

Several nodes along the cytokine–BBB–neuroglia signaling loop represent viable targets for translational intervention. Therapeutic strategies that attenuate upstream cytokine drive, suppress inflammasome activity, modulate the expression of disease-associated microRNAs, and preserve BBB integrity may collectively constitute a comprehensive cardio-neuroprotective framework.

### 7.1. Targeted Cytokine Modulation—Anti-IL-1β and IL-6R Antagonists, and TNF-α Modulators

Clinical evidence supporting the immunomodulatory medications described below derives predominantly from patients with systemic inflammatory diseases, pericarditis, or atherosclerotic cardiovascular disease. Conversely, there is a paucity of randomized trials specifically addressing myocarditis, and the majority of available evidence derives from small cohorts, observational studies, or extrapolation from other inflammatory cardiac conditions.

○ IL-1 pathway. Inhibition of IL-1β (e.g., canakinumab) reduces systemic inflammation and cardiovascular (CV) events, providing proof-of-principle that targeting this cytokine can favorably modify deleterious CV outcomes in humans [109]. The short-acting agent anakinra has been deployed to counter hyperinflammatory states and is mechanistically aligned with myocarditis care pathways involving NLRP3–IL-1β activation (Table 4), [110].
○ IL-6 pathway. IL-6R antagonists, such as tocilizumab, inhibit both classical and trans-signaling. The fusion protein sgp130Fc (olamkicept), which selectively blocks trans-signaling, represents a rational BBB-sparing therapeutic option given the central role of IL-6 in driving endothelial permeability and tight-junction degradation (Table 4), [111].
○ TNF-α pathway. TNF-α mediates leukocyte adhesion, endothelial activation, and matrix remodeling [112]. Although TNF-α neutralization can alleviate inflammation, systemic high-dose blockade worsened heart failure in clinical trials, necessitating caution and advocating for dose- and context-specific application in myocarditis (Table 4), [113].

In established cardiovascular inflammatory conditions, such as atherosclerotic disease and recurrent pericarditis, inhibition of the IL-1 and IL-6 pathways has demonstrated a favorable risk–benefit ratio for reducing cardiac inflammation and improving outcomes. However, randomized controlled trials focused solely on myocarditis are lacking, and current therapeutic approaches rely primarily on extrapolation from broader cardiovascular inflammatory disease data.

Therefore, although cytokine-targeted therapy is mechanistically compelling in viral myocarditis, its clinical use should be considered experimental until myocarditis-specific randomized trial data are obtained.

### 7.2. Inflammasome Inhibitors (NLRP3 Blockade)

NLRP3 senses viral/DAMP signals upstream of caspase-1 activation and IL-1β maturation. The selective small molecule MCC950 significantly blocks NLRP3 and prevents myocardial injury and adverse remodeling in preclinical models [114]. The oral NLRP3 inhibitor dapansutrile (OLT1177) has demonstrated anti-inflammatory and favorable cardiocirculatory effects in early-phase clinical trials, suggesting the feasibility of inflammasome blockade for systemic inflammatory cardiopathies (Table 4), [115].

Clinical implication: NLRP3 blockade may downstream regulate the IL-1 axis and inhibit pyroptosis, potentially reducing both cardiac damage and secondary BBB breakdown (Table 4).

### 7.3. miRNA Therapies (Anti-miR-155, miR-146a Mimics)

miR-155 enhances TLR/NF-κB signaling and suppresses junctional/adhesion proteins (e.g., CLDN-1, ANXA-2, and Suppressor of Cytokine Signaling 1 (SOCS1)), all contributing to endothelial leak and macrophage activation. Anti-miR-155 diminishes vascular permeability and inflammatory infiltration in vivo [116,117]. miR-146a/b counter-regulate TRAF6/IRAK1 to inhibit NF-κB signaling; miR-146a mimics reduce cytokine production and, in a context-dependent manner, modulate aberrant Th17 responses [93,95]. Delivery through exosomes or lipid nanoparticles provides the possibility of specific organ targeting and could potentially cross or recondition the BBB (Table 4), [118].

Clinical implication: miRNA therapeutics have the potential to modulate multiple inflammatory nodes simultaneously; anti-miR-155 and miR-146a mimics are considered leading cardio–neuroinflammatory agents (Table 4).

### 7.4. Neuroprotective Strategies for BBB Stabilization

Protection of the NVU reduces heart-to-brain spillover of inflammation (Table 4):

○ Tight junction and cytoskeleton stabilization: IL-6 trans-signaling blockade; inhibition of RhoA/ROCK (e.g., fasudil) prevents stress-fiber contraction and paracellular leak [119].
○ Endothelial support: activation of angiopoietin-1/Tie2 and Wnt/β-catenin pathways (e.g., Norrin/Wnt7a) restores junctional complexes (claudin-5, occludin) and reduces transcytosis [120].
○ Anti-glial activation: central anti-inflammatory agents (e.g., minocycline) attenuate cytokine-mediated BBB damage and subsequent neurotoxicity [121].

Clinical implication: BBB-targeted therapy may serve as an adjunct to cytokine suppression, breaking the neuroglia–BBB–cytokine loop at the endothelial interface.

### 7.5. Combined Cardio–Neuroprotective Pharmacology

It is anticipated that future care will combine cardiac anti-inflammatory strategies (IL-1/IL-6/NLRP3 inhibition) with BBB conservation and central autonomic regulation. Low-level vagus nerve stimulation (or other neuromodulatory methods) can inhibit sympathetic hyperactivation and diminish cardiac inflammation post-injury [122]. Rational combination therapy—for example, IL-1 blockade + sgp130Fc (to protect the endothelium) ± anti-miR-155 (to stabilize junctions and dampen macrophage activation)—targets different levels of the loop to achieve synergistic benefit (Table 4).

Priority next steps include:

○ Biomarker-based patient selection (IL-6/CRP, EV-miR-155/miR-146a, endothelial permeability markers);
○ Adaptive endpoints: cardiac measures (left ventricular ejection fraction (LVEF), global longitudinal strain (GLS), arrhythmia burden) and neurovascular assessments (BBB permeability imaging, neurocognitive scores);
○ Multi-point inhibition platform trials (Table 4).

### 7.6. Translational Limitations and Barriers to Clinical Implementation

Despite compelling mechanistic rationale, it remains challenging to translate cytokine- and inflammasome-targeted therapies into validated treatments for viral myocarditis. The following factors account for the gap between experimental promise and clinical validation.

First, myocarditis is a heterogeneous clinical syndrome that includes viral-positive, virus-negative autoimmune, toxic, and immune checkpoint inhibitor-associated forms, each with a unique immunopathogenic profile. Clinical experience indicates that immunosuppressive therapy may be advantageous in specific cases of virus-negative inflammatory cardiomyopathy, especially when informed by endomyocardial biopsy and the absence of viral persistence, as evidenced by controlled studies of immunosuppression in inflammatory cardiomyopathy (e.g., prednisone plus azathioprine) [123]. However, this strategy is not applicable to active viral myocarditis, as the suppression of antiviral immunity could theoretically hinder viral clearance.

Second, a significant portion of the clinical evidence endorsing IL-1 pathway inhibition originates from generalized cardiovascular inflammatory diseases rather than myocarditis-specific randomized trials. The CANTOS trial demonstrated that canakinumab-mediated IL-1β blockade reduces the risk of recurrent cardiovascular events in patients with atherosclerotic disease and elevated inflammatory markers, providing proof-of-principle that targeting upstream inflammation can improve cardiovascular outcomes [109]. Likewise, IL-1 inhibition with anakinra has demonstrated favorable outcomes in diverse cardiovascular inflammatory conditions, such as pericarditis and inflammatory heart failure [124]. However, these data predominantly derive from non-myocarditis populations, necessitating caution in their extrapolation to viral myocarditis.

Third, contemporary management of myocarditis prioritizes etiologic stratification and individualized treatment rather than uniform cytokine suppression. Current clinical frameworks emphasize the significance of biopsy-guided diagnosis, viral genome detection, and tailored immunomodulation strategies, indicating that therapeutic decisions are largely contingent upon viral persistence status and inflammatory phenotype [125]. This precision-based approach further complicates the design of large randomized trials.

Fourth, the timing of therapeutic intervention remains critical. Anti-inflammatory therapy during early antiviral defense may diminish protective immune responses, whereas delayed intervention may allow detrimental remodeling to progress. Moreover, spontaneous recovery occurs in a significant proportion of myocarditis patients, complicating endpoint selection and the statistical power of interventional trials.

Finally, validated biomarkers reflecting real-time heart–brain axis activity are lacking. Circulating IL-6, CRP, and extracellular vesicle-associated microRNAs may serve as indirect markers of systemic inflammation; however, no standardized clinical metric currently exists that accurately quantifies neurocardiac inflammatory coupling. This limitation impairs both patient selection and therapeutic monitoring in cardio-neuroinflammatory settings.

In summary, these translational barriers collectively help explain why therapies demonstrating robust preclinical efficacy have yet to achieve definitive validation in viral myocarditis-associated neuroinflammation.

## 8. Conclusions

Viral myocarditis exemplifies how localized cardiac infection can propagate systemic inflammation that extends to the brain. The IL-1β/NLRP3, IL-6, TNF-α, and IL-17/IL-23 cytokine pathways, together with regulatory microRNAs and extracellular vesicle (EV) signaling, constitute a self-reinforcing feedback loop that amplifies innate immune activation across cardiac and neural tissues, thereby converting an initially localized viral etiology into multiorgan inflammatory comorbidities. In myocarditis, the heart and brain function as an integrated pathophysiological system rather than as independently affected organs. This axis remains persistently active and underlies arrhythmias, progressive cardiac remodeling, cognitive decline, and worsening heart failure. Therapeutics that modulate cytokines, inhibit inflammasomes, target microRNAs, stabilize the endothelium, and recalibrate the autonomic nervous system represent promising strategies to disrupt this self-sustaining cycle. Future research should prioritize the validation of biomarkers across diverse patient cohorts, the application of advanced imaging modalities to assess blood–brain barrier integrity, and the development of more refined translational tools. Together, these advances will strengthen the cardio-neuroprotective framework needed to better safeguard both organs. If viral myocarditis is viewed as a disorder of the systemic inflammatory axis rather than merely a cardiac condition, it may fundamentally transform clinical approaches to the diagnosis and treatment of inflammatory cardiovascular disease.

## Figures and Tables

**Table 1:** Comparative Features of Major Viral Etiologies in Heart–Brain Axis Activation.

Virus	Primary Cardiac Target	Dominant Mechanism	Systemic/Brain Effects	Distinct Heart–Brain Features
Enteroviruses (CVB3)	Cardiomyocytes	Direct cytolysis + TLR3/NF-κB activation	Cytokine surge, IL-1β/NLRP3 activation	Strong inflammasome-driven neuroinflammation
Parvovirus B19	Endothelial cells	Endothelial dysfunction, microvascular inflammation	Microvascular hypoperfusion, BBB vulnerability	Vascular-mediated heart–brain coupling
SARS-CoV-2	Endothelium + pericytes	ACE2-mediated injury, immune dysregulation, microthrombosis	Cytokine storm, coagulopathy, BBB leak	Prominent thromboinflammatory neurovascular injury
HHV-6	Latent cardiomyocyte infection	Chronic low-grade immune activation	Persistent cytokine elevation	Potential chronic neuroinflammatory sensitization

Note: Although downstream cytokine amplification is shared, viral etiologies differ in primary cellular targets and vascular involvement, which may influence heart–brain axis dynamics and therapeutic considerations.

**Table 2:** Key cytokines involved in heart–brain axis signaling in viral myocarditis.

Molecule	Primary Source(s)	Effects in the Heart	Effects in Brain/BBB	Key Pathways
IL-1β	Activated macrophages, monocytes, cardiomyocytes, microglia	Promotes cardiomyocyte dysfunction, contractile impairment, fibrosis, and adverse remodeling	Increases BBB permeability, activates microglia and astrocytes, promotes neuroinflammation	NLRP3 inflammasome, NF-κB, MAPK
IL-6	Cardiomyocytes, endothelial cells, macrophages, fibroblasts	Induces hypertrophy, fibrosis, and inflammatory amplification	Disrupts BBB integrity via tight-junction destabilization; promotes glial activation	IL-6R/gp130 → JAK/STAT3
TNF-α	Macrophages, T cells, cardiomyocytes, endothelial cells	Promotes apoptosis, necroptosis, ECM remodeling, arrhythmogenesis	Induces BBB disruption, glial activation, excitotoxicity	TNFR1/TNFR2, NF-κB, RhoA/ROCK
IL-17 (Th17-derived)	Th17 lymphocytes, γδ T cells	Enhances neutrophil recruitment, myocardial injury, fibrosis	Increases BBB permeability, promotes microglial IL-1β release	IL-17R, STAT3, NF-κB
IL-23	Macrophages, dendritic cells	Sustains Th17 responses, amplifies myocardial inflammation	Promotes neuroinflammation indirectly via Th17 maintenance	IL-23R → STAT3
IL-33	Endothelial cells, fibroblasts, astrocytes	Context-dependent: cardioprotective (anti-fibrotic) or pro-inflammatory if chronically elevated	Activates microglia, increases cytokine release, contributes to BBB dysfunction	ST2L → MyD88/TRAF6/NF-κB

**Table 3:** Regulatory microRNAs involved in heart–brain axis modulation in viral myocarditis.

miRNA	Validated/Functional Targets	Cardiac Effect	BBB Effect	Therapeutic Potential
miR-155	SOCS1, CLDN1, ANXA2, SHIP1	Amplifies myocardial inflammation, promotes immune cell activation, worsens myocarditis severity and cardiac dysfunction	Disrupts tight junction integrity, increases endothelial permeability, promotes leukocyte infiltration	Anti-miR-155 therapy may attenuate inflammation, stabilize BBB, and improve cardio–neuro outcomes
miR-146a/b	TRAF6, IRAK1, NF-κB signaling components	Limits excessive inflammatory signaling, exerts cardioprotective and anti-fibrotic effects	Preserves BBB integrity by dampening endothelial and glial inflammatory activation	miR-146a/b mimics represent a potential strategy to suppress chronic inflammation and restore homeostasis

**Table 4:** Therapeutic targets and translational strategies for modulation of the heart–brain axis in inflammatory cardiac disease.

Target	Strategy	Drug/Approach	Evidence Level	Effect on Heart–Brain Axis
IL-1β	Cytokine neutralization	Anakinra, Canakinumab	Clinical (approved in systemic inflammatory diseases; limited myocarditis-specific data; extrapolated cardiovascular evidence)	Reduces systemic inflammation, attenuates myocardial injury, may limit BBB disruption and neuroinflammation
IL-6	IL-6R blockade/trans-signaling inhibition	Tocilizumab; sgp130Fc	Clinical (approved in systemic inflammatory diseases; limited myocarditis-specific data; extrapolated cardiovascular evidence)	Dampens cytokine amplification, improves endothelial and BBB stability, reduces neuroinflammatory signaling
NLRP3 inflammasome	Direct inflammasome inhibition	MCC950; OLT1177 (dapansutrile)	Preclinical; early clinical (OLT1177)	Suppresses IL-1β/IL-18 production, limits myocardial inflammation, protects BBB integrity
miR-155	miRNA inhibition	Anti-miR-155 oligonucleotides	Preclinical	Reduces immune hyperactivation, stabilizes endothelial junctions, improves cardio–neuro inflammatory balance
RhoA/ROCK	Kinase inhibition	Fasudil	Clinical (approved in some countries for vascular disorders)	Improves endothelial function, preserves BBB integrity, reduces sympathetic-driven vascular and cardiac dysfunction
BBB integrity	Barrier stabilization/endothelial protection	Angiopoietin-1 mimetics, antioxidants, endothelial-protective agents	Preclinical	Limits leukocyte infiltration and cytokine penetration into CNS, attenuating neuroinflammation and autonomic disruption
Autonomic imbalance	Neuromodulation	Vagus nerve stimulation (VNS)	Clinical (approved for epilepsy/depression; emerging cardiac data)	Restores sympatho–vagal balance, reduces systemic inflammation, protects against arrhythmias and adverse remodeling

Note: Evidence levels reflect overall clinical development status. While IL-1 and IL-6 inhibitors are approved and supported by clinical trials in systemic inflammatory and selected cardiovascular conditions, myocarditis-specific randomized controlled trials remain limited, and current therapeutic positioning in viral myocarditis is largely extrapolated from data derived from broader cardiovascular inflammatory disease studies.

## Data Availability

No new data were created or analyzed in this study. Data sharing is not applicable to this article.

## Abbreviations

The following abbreviations are used in this manuscript:

ACE2	Angiotensin-Converting Enzyme 2
Akt	Protein Kinase B
Ang-1	Angiopoietin-1
ANS	Autonomic Nervous System
ANXA-2	Annexin A2
BRS	Baroreflex Sensitivity
BBB	Blood–Brain Barrier
CAR	Coxsackievirus–Adenovirus Receptor
CAs	Catecholamines
CCL2	C–C Motif Chemokine Ligand 2
CLDN-1	Claudin-1
CNS	Central Nervous System
COX-2	Cyclooxygenase-2
CRP	C-Reactive Protein
CV	Cardiovascular
CVB3	Coxsackievirus B3
CXCL10	C–X–C Motif Chemokine Ligand 10
DAMP	Damage-Associated Molecular Patterns
DCM	Dilated Cardiomyopathy
DMVN	Dorsal Motor Nucleus of the Vagus
EC	Endothelial Cell
ECM	Extracellular Matrix
EV	Extracellular Vesicle
FAK	Focal Adhesion Kinase
GLS	Global Longitudinal Strain
GP130	Glycoprotein 130
HF	Heart Failure
HHV-6	Human Herpesvirus 6
ICAM-1	Intercellular Adhesion Molecule 1
IFN	Interferon
IL	Interleukin
IL-6R	Interleukin-6 Receptor
IRAK1	Interleukin-1 Receptor-Associated Kinase 1
IRF	Interferon Regulatory Factor
JAK	Janus Kinase
JAK2	Janus Kinase 2
JNK	c-Jun N-terminal kinase
LVEF	Left Ventricular Ejection Fraction
MCC950	Selective NLRP3 Inflammasome Inhibitor
miR	microRNA
MMP	Matrix Metalloproteinase
MyD88	Myeloid Differentiation Primary Response 88
NA	Nucleus Ambiguus
NF-κB	Nuclear Factor Kappa B
NLRP3	NOD-, LRR- and Pyrin Domain-Containing Protein 3
NTS	Nucleus Tractus Solitarius
NVU	Neurovascular Unit
OLT1177	Dapansutrile (NLRP3 inhibitor)
PAMP	Pathogen-Associated Molecular Pattern
PDGF-β	Platelet-Derived Growth Factor Beta
PI3K	Phosphoinositide 3-Kinase
PVN	Paraventricular Nucleus
PGE2	Prostaglandin E2
RhoA	Ras Homolog Family Member A
ROCK	Rho-Associated Protein Kinase
RORγt	Retinoic Acid-Related Orphan Receptor Gamma t
RVLM	Rostral Ventrolateral Medulla
SARS-CoV-2	Severe Acute Respiratory Syndrome Coronavirus 2
sIL-6R	Soluble Interleukin-6 Receptor
SOCS1	Suppressor of Cytokine Signaling 1
STAT3	Signal Transducer and Activator of Transcription 3
ST2L	Suppression of Tumorigenicity 2 Ligand
TGF-β	Transforming Growth Factor Beta
Th17	T Helper 17 Cells
Tie2	Tyrosine Kinase with Immunoglobulin-like and EGF-like Domains 2
TJ	Tight Junction
TLR	Toll-Like Receptor
TNF	Tumor Necrosis Factor
TNFR	Tumor Necrosis Factor Receptor
TRAF6	TNF Receptor Associated Factor 6
TRIF	TIR-domain-containing Adaptor Inducing Interferon-β
VCAM-1	Vascular Cell Adhesion Molecule 1
Wnt	Wingless-related Integration Site
ZO-1	Zonula Occludens-1
